# Clinical and Genetic Spectrum of a Large Cohort of Patients With Leukocyte Adhesion Deficiency Type 1 and 3: A Multicentric Study From India

**DOI:** 10.3389/fimmu.2020.612703

**Published:** 2020-12-16

**Authors:** Priyanka Madhav Kambli, Umair Ahmed Bargir, Reetika Malik Yadav, Maya Ravishankar Gupta, Aparna Dhondi Dalvi, Gouri Hule, Madhura Kelkar, Sneha Sawant-Desai, Priyanka Setia, Neha Jodhawat, Nayana Nambiar, Amruta Dhawale, Pallavi Gaikwad, Shweta Shinde, Prasad Taur, Vijaya Gowri, Ambreen Pandrowala, Anju Gupta, Vibhu Joshi, Madhubala Sharma, Kanika Arora, Rakesh Kumar Pilania, Himanshi Chaudhary, Amita Agarwal, Shobita Katiyar, Sagar Bhattad, Stalin Ramprakash, Raghuram CP, Ananthvikas Jayaram, Vinod Gornale, Revathi Raj, Ramya Uppuluri, Meena Sivasankaran, Deenadayalan Munirathnam, Harsha Prasad Lashkari, Manas Kalra, Anupam Sachdeva, Avinash Sharma, Sarath Balaji, Geeta Madathil Govindraj, Sunil Karande, Ruchi Nanavati, Mamta Manglani, Girish Subramanyam, Abhilasha Sampagar, Indumathi CK, Parinitha Gutha, Swati Kanakia, Shiv Prasad Mundada, Vidya Krishna, Sheela Nampoothiri, Sandeep Nemani, Amit Rawat, Mukesh Desai, Manisha Madkaikar

**Affiliations:** ^1^ Center of Excellence for PIDs, Department of Pediatric Immunology and Leukocyte Biology, Indian Council of Medical Research- National Institute of Immunohaematology, Mumbai, India; ^2^ Department of Immunology, Bai Jerbai Wadia Hospital for Children, Mumbai, India; ^3^ Department of Bone Marrow Transplant, Bai Jerbai Wadia Hospital for Children, Mumbai, India; ^4^ Allergy Immunology Unit, Department of Pediatrics, Advanced Pediatrics Centre, Post Graduate Institute of Medical Education and Research, Chandigarh, India; ^5^ Department of Clinical Immunology & Rheumatology, Sanjay Gandhi Postgraduate Institute, Lucknow, India; ^6^ Department of Pediatric Immunology and Rheumatology, Aster CMI Hospital, Bengaluru, India; ^7^ Pediatric Hemat-Oncology and Bone Marrow Transplant Unit, Aster CMI Hospital, Bengaluru, India; ^8^ Department of Hematology and Pathology, Neuberg Anand Diagnostic and Research Centre, Bangalore, India; ^9^ Department of pediatric, Indira Gandhi Institute of Child Health, Bangalore, India; ^10^ Department of Pediatric Hematology, Oncology, Blood and Marrow Transplantation, Apollo Hospitals, Teynampet, India; ^11^ Department of Pediatric, Hemato-oncology, Kanchi Kamakoti Childs Trust Hospital, Chennai, India; ^12^ Department of Paediatrics, Kasturba Medical College, Mangalore, Manipal Academy of Higher Education, Manipal, India; ^13^ Department of Pediatric Hematology Oncology BMT, Sir Ganga Ram Hospital, New Delhi, India; ^14^ Dr. Rajendra Prasad Government Medical College, Tanda, India; ^15^ Department of Paediatrics, Institute of Child Health and Hospital for Children, Chennai, India; ^16^ Department of Paediatrics, Calicut Medical College, Medical College Junction, Kozhikode, India; ^17^ Department of Pediatrics, King Edward Memorial Hospital, Mumbai, India; ^18^ Department of Neonatology, King Edward Memorial Hospital, Mumbai, India; ^19^ Department of Pediatric, Oncology, Hematology & BMT, Comprehensive Thalassemia Care Center and Bone Marrow, Mumbai, India; ^20^ Department of Pediatrics, Colours Children Hospital, Nagpur, India; ^21^ Department of Pediatrics, KIES Dr. Prabhakar Kore Hospital & Medical Research, Belgaum, India; ^22^ Department of Pediatrics, St. John’s Medical College, Bengaluru, India; ^23^ Department of Paediatric Haematology and Oncology, Little Stars Children’s Hospital, Hyderabad, India; ^24^ Department of Hematology-Oncology, Lilavati Hospital and Research Centre, Mumbai, India; ^25^ Department of Pediatrics, Mundada hospital, Latur, India; ^26^ Department of Pediatrics, Sri Ramachandra Medical College, Chennai, India; ^27^ Department of Pediatric Genetics, Amrita Institute of Medical Science & Research Center, Cochin, India; ^28^ Nihira Diagnostic Lab, Arihant Galaxy, Ganesh Naga, Sangli, India

**Keywords:** Leukocyte Adhesion deficiency, CD18, CD11, *FERMT3*, *ITGβ2*

## Abstract

Leukocyte adhesion deficiency (LAD) syndrome is a group of inborn errors of immunity characterized by a defect in the cascade of the activation and adhesion leading to the failure of leukocyte to migrate to the site of tissue injury. Three different types of LAD have been described. The most common subtype is LAD type 1 (LAD1) caused due to defects in the *ITGβ2 *gene. LAD type 2 (LAD2) is caused by mutations in the *SLC35C1* gene leading to a generalized loss of expression of fucosylated glycans on the cell surface and LAD type 3 (LAD3) is caused by mutations in the *FERMT3* gene resulting in platelet function defects along with immunodeficiency. There is a paucity of data available from India on LAD syndromes. The present study is a retrospective analysis of patients with LAD collated from 28 different centers across India. For LAD1, the diagnosis was based on clinical features and flow cytometric expression of CD18 on peripheral blood leukocytes and molecular confirmation by Sanger sequencing. For patients with LAD3 diagnosis was largely based on clinical manifestations and identification of the pathogenic mutation in the *FERMT3* gene by next-generation Sequencing. Of the total 132 cases diagnosed with LAD, 127 were LAD1 and 5 were LAD3. The majority of our patients (83%) had CD18 expression less than 2% on neutrophils (LAD1°) and presented within the first three months of life with omphalitis, skin and soft tissue infections, delayed umbilical cord detachment, otitis media, and sepsis. The patients with CD18 expression of more than 30% (LAD1^+^) presented later in life with skin ulcers being the commonest manifestation. Bleeding manifestations were common in patients with LAD3. Persistent neutrophilic leukocytosis was the characteristic finding in all patients. 35 novel mutations were detected in the *ITGβ2* gene, and 4 novel mutations were detected in the *FERMT3* gene. The study thus presents one of the largest cohorts of patients from India with LAD, focusing on clinical features, immunological characteristics, and molecular spectrum.

## Introduction

Leukocyte adhesion deficiency (LAD) is a rare phagocytic disorder characterized by a defect in the trafficking of leukocytes from the blood vessels to the site of tissue injury (1–4). These patients usually present in infancy with delayed separation of the umbilical cord, omphalitis, and necrotic infections of the skin and mucosal surfaces (5). The absence of pus and persistent marked neutrophilic leukocytosis are the hallmarks of LAD. Three types of LAD have been described, with LAD type 1 (LAD1) being the most common form. LAD1 is caused due to defect in the *ITGβ2* gene encoding the common beta subunit of β2 integrins (CD18) (5–9). β2 integrins form a heterodimer by non-covalently binding to the different subunits including, αL (CD11a), αM (CD11b), αX (CD11c), and αD (CD11d) (1, 4, 9). In a setting of strong clinical suspicion, the immunological workup for diagnosis of LAD1 involves studying the flow cytometric expression of CD18 and CD11 on leukocytes followed by molecular confirmation. Depending on the CD18 expression on neutrophils LAD1 patients are classified into severe (CD18 expression <2%), moderate (2%–30%), and mild (>30%) (1, 2, 5, 10). LAD type 2 (LAD2) is caused due to mutations in the *SLC35C1* gene leading to defective expression of cell surface fucosylated glycan structures (11, 12). These patients suffer from recurrent bacterial infections, severe mental, and growth retardation characterized by distinct facial characteristics (12). Flow cytometry demonstrates the absence of SLeX (CD15a) expression on cell-surface glycoproteins, along with deficiency of H antigen on erythroid cells, resulting in the Bombay phenotype (13). LAD type 3 (LAD3) is caused by a mutation in the *FERMT3* gene that encodes protein kindlin-3 which plays a crucial in integrin activation (14–16). These patients also have severe recurrent bacterial infections, persistent leukocytosis, and delayed umbilical cord fall with a platelet aggregation defect that results in severe bleeding manifestation (17). Though individual case reports and small case series are available from India (18–23), there is a paucity of data on the clinical, immunological, and molecular spectrum in LAD. In this study, we report a retrospective cohort study of 132 patients LAD patients from 28 different centers of India.

## Materials and Methods

Patients with a clinical suspicion of LAD referred to the Indian Council of Medical Research-National Institute of Immunohaematology (ICMR-NIIH) and other tertiary care centers in India between 1990 and 2020 were retrospectively analyzed in this study. The clinical and laboratory information about the age of presentation, age at diagnosis, site of infections, organisms isolated, umbilical cord complications, family history, consanguinity, complete blood count (CBC), immunological investigations were collected from the data available. The study was approved by the institutional ethics committee of ICMR-NIIH.

As a part of the diagnostic workup of LAD the flow cytometric expression of CD18, CD11 markers on leukocytes was assessed. Based on the CD18 expression on neutrophils, LAD1 was sub-classified into three phenotypes viz. severe (LAD1°) with CD18 expression <2% moderate (LAD1^-^) phenotype with CD18 expression 2%–30%, and mild (LAD1^+^) phenotype with CD18 expression ≥30%. CD11a expression data were available for 89 patients. We analyzed our data by looking at different parameters like median fluorescence intensity (MFI) and stain index (SI) index on different populations of leukocytes via, neutrophils, lymphocytes, and monocytes.

Molecular confirmation was performed was done using Sanger sequencing for LAD1 and next-generation sequencing (NGS) for LAD3. The candidate variants identified by NGS were confirmed by Sanger sequencing in the index and family members.

Graph pad prism version 5.03 statistical software was used to perform statistical analysis. The descriptive variables were expressed as percentage counts and the median-interquartile range (IQR) were used. The groups in this study were compared using the one-way ANOVA. The test was performed at a 95% confidence interval (95% CI), and p<0.05 was considered statistically significant. Kaplan–Meier evaluation was used to predict the survival probabilities of the patients.

## Results

### Patients Characteristics

In this study, we analyzed a total of 127 cases from 125 families with LAD1 and 5 cases from 4 families with LAD3. LAD2 were not reported in our cohort. The clinical and demographic features of the patients are as shown in **Table 1**. Consanguinity was seen in 51% of the patients. Male preponderance was seen in our cohort (62%). The genetic diagnosis was available in 80% of cases (n=105) cases. White blood count (WBC) and absolute neutrophil count (ANC) were noted in all the cases with a median of 53 x10^3^/μl (14–167 x10^3^/all) and 36 x10^3^/μl (22–137x10^3^/μl), respectively. It was observed that the ANC was higher in LAD1° 40 × 10^3^/μl (11–136× 10^3^/μl) cases as compared to LAD1**^-^** 25 × 10^3^/μl (16–74× 10^3^/μl), LAD1^+^ 27 × 10^3^/μl (10–91× 10^3^/μl), and LAD3 21 × 10^3^/μl (10–38× 10^3^/μl).

**Table 1:** Clinical characteristics of patients with leukocyte adhesion deficiency (LAD).

	LAD1°		LAD1^-^		LAD1^+^		LAD3	
Total patient (n)	106		11		10		5	
Age of presentation in months Median (Range)	0.3 (0.03–12)		1 (0.09–72)		2.5 (0.5–132)		0.8 (0.03–1)	
Age of diagnosis in months Median (Range)	3 (0.1–48)		5 (1–78)		84 (3–168)		34 (3–168)	
Median WBC count (Range)	57 (16.5–167)		38.6 (19.2–140)		32.5 (14.7–102)		30 (18–99.6)	
Median ANC × 10^3^/μl (Range)	40 (11–136)		25 (16–74)		27 (10–91)		21 (10–38)	
	n	%	n	%	n	%	n	%
Gender (Males)	63	59	7	63	8	80	4	80
Family History	38	36	1	9	3	30	2	40
Consanguinity	54	51	5	45	5	50	2	40
Umbilical cord complication	65	61	7	64	2	20	3	60
LRTI	43	41	5	45	3	30	–	–
Sepsis	39	37	3	27	2	20	–	–
Skin infections	55	52	4	36	7	70	–	–
Periodontal infections	11	10	–	–	–	–	1	20
Otitis media	22	21	2	18	1	10	1	20
Diarrhea	9	8.5	1	9	–	–	–	–
Failure to thrive	31	29	3	27	4	40	–	–
Meningitis	5	5	–		–		–	–
Bleeding Manifestation	–		–		–		5	100

### Clinical and Genetic Characteristics of Leukocyte Adhesion Deficiency 1

On the basis of absent or abnormal CD18 expression on the surface of neutrophils, three distinct phenotypes were observed in our cohort. The commonest being LAD1° seen in 83% (n=106) individuals, followed by LAD1^-^ phenotype seen in 9% (n=11) of the cases and LAD1^+^ in 8% (n=10). The mean CD18 expression in LAD1^-^ and LAD1^+^ patients was 9.6 ± 5% (3%–18%) and 68 ± 24% (32%–99%), respectively. The median fluorescence intensity was noted in 76/127 LAD1 patients. **Figure S1**, presents the percentage, MFI, and SI on neutrophils, monocytes, and lymphocytes in LAD1°, LAD1^-^, and LAD1^+^ cases. Although the expression of CD18 was >30% in LAD1^+^ cases, the CD11a was significantly reduced in 80% (8/10) of the cases. The median age of presentation was 0.3 months (0.03–12 months) for the LAD1° patients, 1 month (0.09–72 months) for LAD1^-^ and 2.5 months (0.5–132 months) for patients with LAD1^+^. The median age of diagnosis was 3 months (0.1–48 months) for LAD1°, 5 months (1–78 months) for LAD1^-^ and 84 months (3–168 months) for LAD1^+^.

Umbilical cord related complications like omphalitis (64%) and delayed separation (62%) were the most common manifestation seen in the LAD1° and LAD1^-^ cases. Other frequent infections included lower respiratory tract infection (LRTI) in 41% (43/106), sepsis in 37%. Necrotic skin ulcer was the most common infection in LAD1^+^ which may mimic pyoderma gangrenosum. The perianal region was the commonest site in LAD1^-^ cases (27%). Infectious organisms were isolated from 69 cases comprising predominantly bacterial infections including *Pseudomonas aeruginosa* (n=28), *Staphylococcus aureus* (n=17), and *Klebsiella pneumonia* (n=11), and fungal infections were noted in 7 patient. Also, unusual organisms like *Proteus* sp.*, Citrobacter* sp.*, Stingomonas paucimobilis, and Acinetobacter baumani* were noted in a few patients.

**Figure 1:**
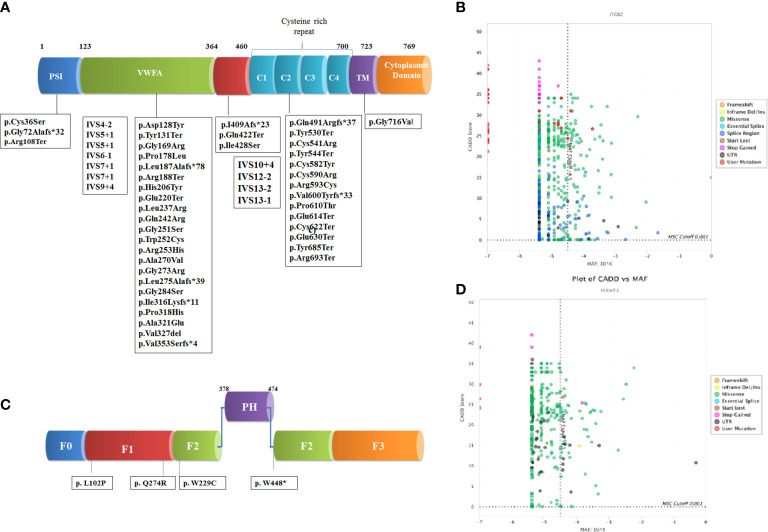
Schematic representation of domain wise distribution of the mutations identified in our LADpatients **(A)** *β2* integrin **(B)** kindlin-3 protein. Combined annotation–dependent depletion (CADD) and Minor allele frequency (MAF) scores of the variants reported in gnomAD and novel variants identified in our cohort for the genes **(C)** *ITGβ2* and **(D)** *FERMT3* using PopViz software (24). The mutation significance cutoff (MSC) with 99% confidence interval is shown in dotted line.

Direct Sanger sequencing of the *ITGβ2* gene revealed 57 disease-causing variants in 105 patients (**Table S2**), including 30 patients we have previously reported (18). These mutations were clustered mostly in exons 6 (22%) and exon 7 (11%). The spectrum of mutation has been shown in **Figure 1**. The frequency of mutation c.533C>T (p.Pro178Leu) and c.817G>A (p.Gly273Arg) was high. The majority of the patients (n=95) had homozygous mutations, while compound heterozygous mutations were identified in only 10 patients. These compound heterozygous mutations were seen only LAD1° cases. Missense mutations (40%) were the most common mutations identified in our cohort followed by nonsense (21%), splice site (19%), and frameshift (19%). 54% of the mutations were located in exon 5–9, a highly conserved region of the extracellular domain of CD18 followed by cysteine-rich repeat region (CRR) domain (32%), Mid region (7%), plexins, semaphorins, and integrins domain (PSI) domain (5%) and transmembrane domain (TM) region (2%). Missense and nonsense mutations were frequently seen in LAD1° and LAD1^-^ patients. On the other hand, splice site mutations affected almost 60% of LAD1^+^ patients.

### Clinical and Genetic Features of Leukocyte Adhesion Deficiency 3

Recurrent infections and severe bleeding manifestation were seen in all patients within 1 month of life. The median age of diagnosis was 34 months (3–168 months). Omphalitis was seen in 3/5 patients. CD18 expression on the surface of the leukocytes was normal in all patients. The molecular diagnosis using NGS technology identified four novel pathogenic variants in the *FERMT3* gene in five LAD3 patients which were conserved across the species. Out of these 3 were missense mutations and one was nonsense mutations.

### Outcome

**Figure 2:**
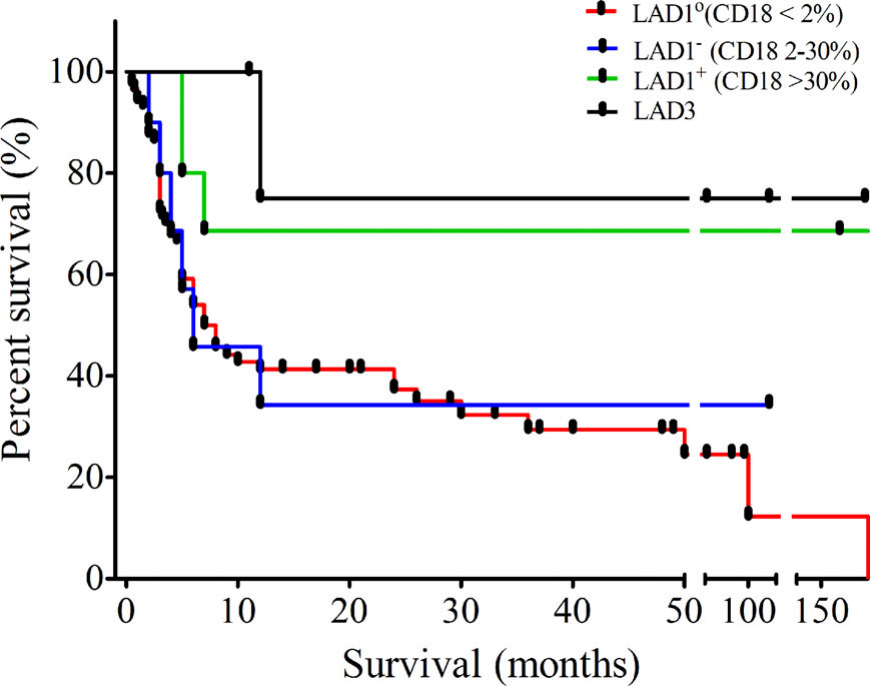
Outcome of leukocyte adhesion deficiency (LAD) patients: Kaplan-Meier curve showing survival of patients diagnosed with LAD in our cohort.

Follow up data were available for 124 patients with the median follow up duration of 7 months (0.033–216 months), 6 months (2–120 months), 182 months (5–276 months), and 60 months (11–192 months) for LAD1°, LAD1^-^, LAD1^+^, and LAD3, respectively. Out of these, 81% of patients expired due to severe infections in absence of hematopoietic stem cell transplant (HSCT). The majority of them died within the first year of life (n=70). The overall survival in our LAD1 cohort is only 14% while that of LAD3 is 83%. Mortality was higher in LAD1° patients with only 6% survival beyond 2 years as compared to 16% of the LAD1^-^ and 60% of the LAD1^+^ patients in our cohort (**Figure 2**). Twelve patients underwent HSCT. Of these, ten patients underwent stem cell transplant (SCT) from HLA-matched related donors, whereas two patients received graft from matched unrelated donor. Three patients expired due to severe graph versus host disease (GVHD) and secondary complications.

## Discussion

Leukocyte adhesion deficiency is a rare phagocytic disorder associated with defective neutrophil recruitment, rolling, and adhesion (1, 25–27). To the best of our knowledge, this is the largest comprehensive study on clinical, immunological, and molecular findings of LAD from India. Most of our patients presented with recurrent bacterial infections with a history of umbilical cord related complications. The infective spectra was similar to other reported cohorts with LRTI and sepsis being the commonest (5, 8, 9, 28–37). Delayed umbilical cord fall was seen in only 66% of the cases. Periodontal infections including gingivitis and oral ulcers are reported in 24% of severe and 52% of moderate LAD1 patients (5). In our cohort, it was observed in 10% of LAD1° patients. This might be because of under-diagnosis of mild LAD1 phenotype. Late- onset autoimmune complications have been reported previously (38). However, it was not observed in our patients as most of them expired in infancy.

The degree of severity of infections in LAD1° and LAD1^-^ patients was high with poor survival of 8% and 33% (beyond 24 months), respectively. On the other hand, patients with LAD1^+^ had a more variable clinical phenotype with a significant difference between the age of onset and diagnosis. We observed that the mortality rate was high in LAD1° as well as LAD1^-^ compared to LAD1^+^.

The clinical suspicion was strengthened with the presence of marked neutrophilic leukocytosis observed in all patients with total WBC count >25 x 10^3^/μl in 86% cases and ANC of >15 x 10^3^/μl in 82% of the cases. The median WBC count was higher in LAD1° as compared to the other subtypes and LAD3 patients, however, there was no significant correlation (r<0.1) between the WBC count and CD18 expression on neutrophil for the entire LAD1 cohort and the three phenotype as seen in the earlier studies (**Figure S2**).

The diagnosis of LAD1 often relies on the percentage of positive neutrophils expressing CD18. For severe forms of LAD1 where the expression is <2% diagnosis is easy and reliable. The expression may vary from patient to patient despite the same underlying disease-causing mutation (5, 30). It was observed that positive predictive value (PPV) of the assay significantly increased from 98.51 to 100% when the MFI of patients and healthy controls are compared (p<0.001) (data not shown). It is known that α- subunit of LFA-1 cannot be efficiently expressed unless it first associates with the β subunit. Previous studies have also reported that the expression of CD11a is abnormal in all the patients of LAD1 irrespective of CD18 expression (7, 32, 37). In P20 and P28, the CD18 expression was 50 & 90% and CD11a expression was 70 & 50%, respectively; SI observed was comparatively below laboratory lower limits obtained from the SI of healthy controls (SI of CD18/CD11a: P20- 1.81/1.59 & P28- 1.61/1.75). This concurs that the addition of the CD11a marker to the assay may increase the diagnostic accuracy (32).

We identified 57 mutations in 105 patients of which 35 were novel suggesting heterogeneity in the mutation spectrum for LAD1. 51% (29/57) of the mutations were identified in the VWFA domain followed by 16% (n=9) in cysteine-rich region and 3% (n=2) PSI domain, 4% (n=2) mid-region and 2% (n=1) in TM. The common mutation identified included c.533C>T (n=9), c.817G>A (n=9), c.751G>A (n=5), c.1224+4A>G (n=5), and c.2077C>T (n=5) in different domains. Out of the total mutations identified in the VWFA domain, 89% of them resulted in absent expression of CD18 on PMNs causing severe infection in LAD1° and LAD1^-^ cases.

50% of LAD1^+^ had a splice site mutation. These patients presented later in life with recurrent skin lesions like pyoderma gangrenosum. All of them had the same c.1224+4A>G mutation and 4 of these patients have been reported by us earlier (39). In contrast to other patients with pyoderma gangrenosum, there is a paucity of neutrophils in the dermis of the skin lesions in patients with LAD1^+^ and they also show only partial and temporary response to steroids. Though the pathogenesis of these inflammatory lesions is not clear, partial expression of CD18 resulting in an aberrant oscillation of integrins on the neutrophil surface and Th17 mediated aberrant inflammatory response may be responsible for these inflammatory skin lesions (40, 41).

LAD3 was diagnosed in 5 patients classically presenting with recurrent infection of skin, ear, and mucosal surfaces, and bleeding from gums and skin. There was a significant variation in the age of diagnosis and presentation in these patients. Unfortunately, platelet aggregation studies were not available for these cases. Unlike LAD1, the surface expression of CD18/CD11 expression on leukocytes was normal in these patients. This disorder has mostly been reported in patients of Arab Maltese, Turkish, or African American origin (15, 16, 20, 42, 43).

The overall outcome in our cohort was poor with 81% mortality. The time taken for the patients from the diagnosis to treatment is critical and many patients are lost before they reach the stage of transplant. HSCT was possible in only 12 cases as most of the patients included in the study were from the last decade when limited transplantation facilities were available in India. However, with the increase in the number of HSCT centers, this scenario may change in the near future. Recent advancements in gene therapy for LAD1 may change the course of management (30).

This study describes the clinical and molecular spectrum of a large cohort of patients of LAD from India. It highlights the importance of analyzing MFI and SI of CD11a along with CD18 for accurate diagnosis of LAD1. It reports a large number of previously unreported mutations in the *ITGβ2* and *FERMT3* gene. Knowledge of the nature and frequency of these mutations is not only important for providing accurate diagnosis and genetic counseling to the families but will also help in the future for planning gene editing and gene therapy strategies for these rare genetic disorders.

## Data Availability Statement

The datasets presented in this study can be found in online repositories. The names of the repository/repositories and accession number(s) can be found in the article/**Supplementary Material**.

## Ethics Statement

The studies involving human participants were reviewed and approved by the Indian Council of Medical Research National Institute of Immunohematology. Written informed consent to participate in this study was provided by the participants’ legal guardian/next of kin. Written informed consent was obtained from the minor(s)’ legal guardian/next of kin for the publication of any potentially identifiable images or data included in this article.

## Author Contributions

PK analyzed the data and wrote the manuscript. UB and RM helped in procuring the clinical details and follow-up of the patients. MD, AR, PT, VG, AP, AG, KA RKP, HC, PK, AA, SunK, SagB, SR, RCP, VinG, RR, RU, MSi, DM, HP, MKa, AnuS, AviS, SarB, GG, ShoK, RN, MamM, GS, AbhS, ICK, ParG, SwaK, SPM, VK, ShN, and SNem supervised the management and follow up of the patients. PK, MG, AD, GH, PS, MKe, SS, NJ, NN, AmrD, PalG, ShwS, ShoK, and AJ performed the laboratory investigations for the different cases. PK, PK, MG, MSh, and VJ were involved in the molecular analysis of the different patients. MM supervised the study and reviewed the manuscript. All authors contributed to the article and approved the submitted version.

## Funding

Support from Indian Council of Medical Research to NIIH is gratefully acknowledged.

## Conflict of Interest

The authors declare that the research was conducted in the absence of any commercial or financial relationships that could be construed as a potential conflict of interest.

## Supplementary Material

Supplementary Figure S1 The percentage expression, median fluorescence intensity (MFI), stain index (SI- ratio of intensity of stain/unstain) of CD18 and CD11a on a) Neutrophils b) Lymphocytes, and c) monocytes.

Supplementary Figure S2 Correlation between WBC count and CD18 expression on neutrophils a) all LAD patients b) LAD1° c) LAD1^-^ d) LAD1^+^ e) LAD3.

Supplementary Table S1 Clinical summary of all the patients diagnosed with LAD.

Supplementary Table S2 Mutation spectrum of *ITGβ2* gene and *FERMT3* genes.
