# Supplementary material for: Clinical and Genetic Spectrum of a Large Cohort of Patients With Leukocyte Adhesion Deficiency Type 1 and 3: A Multicentric Study From India
Source: Front Immunol. 2020 Dec 16;11:612703. doi: 10.3389/fimmu.2020.612703 (PMC7772426; doi:10.3389/fimmu.2020.612703)
Supplement: Supplementary Table S1 — Clinical summary of all the patients diagnosed with LAD. [file Table_1.docx]

| **Phenotype** | **Patient** | **Gender** | **Age of onset (months)** | **Age of Diagnosis (months)** | **Umbilical Cord Separation** | **Other clinical presentations** | **Family History** | **Consanguinity** | ***Organisms Isolated*** | **ANC** | **Outcome** |
| --- | --- | --- | --- | --- | --- | --- | --- | --- | --- | --- | --- |
| LAD1^o^ | P1 | ♀ | 2 | 2.5 | Yes | Omphalitis, Sepsis, fever, hepatomegaly | Yes | Yes | *E. coli, oral candidiasis* | 14080 | Expired |
| LAD1^o^ | P2 | ♂ | 8 | 9 | Yes | Omphalitis, perianal abscess, necrotic skin ulcer, recurrent infections | Yes | No | *Streptococcus sp.* | 16230 | Expired |
| LAD1^o^ | P3 | ♀ | 0.3 | 4 | Yes | Omphalitis, otitis media, delayed wound healing | No | No | *S. aureus, P. aeruginosa* | 16400 | Expired |
| LAD1^o^ | P4 | ♀ | 0.3 | 24 | Yes | Omphalitis, skin rash | Yes | No | *-* | 22980 | Expired |
| LAD1^o^ | P5 | ♂ | 0.03 | 0.03 | Yes | Omphalitis, perianal abscess | Yes | No | *Staphylococcus aureus* | 45059 | Expired |
| LAD1^o^ | P6 | ♂ | 0.03 | 3 | Yes | Omphalitis, necrotic skin ulcer | Yes | No | *-* | 28720 | Expired |
| LAD1^o^ | P7 | ♀ | 0.16 | 12 | Yes | Omphalitis, Pneumonia, pyogenic meningitis, bullous impetigo, otitis media, hypo pigmented patches on neck | No | Yes | *Pseudomonas aeruginosa, MRSA* | 21292 | Expired |
| LAD1^o^ | P8 | ♀ | 1.5 | 2 | Yes | Omphalitis, pneumonia, ear discharge, CNS infection skin rash, hepatomegaly | No | Yes | *-* | 14750 | Expired |
| LAD1^o^ | P9 | ♀ | 0.13 | 1 | Yes | Omphalitis, skin rash, otitis media, bulbous lesions, multiple pustules | Yes | Yes | *Acinetobacter sp.* | 57440 | Expired |
| LAD1^o^ | P10 | ♂ | 0.13 | 2.5 | Yes | Omphalitis, sepsis, respiratory tract infection bronchopneumonia | No | Yes | *Pseudomonas aeruginosa* | 34300 | Expired |
| LAD1^o^ | P11 | ♂ | 1 | 5 | Yes | Omphalitis, fever, cough, breathlessness, respiratory tract infection, hypo-pigmentation on cheek & neck | Yes | Yes | *-* | 96256 | Expired |
| LAD1^o^ | P12 | ♂ | 3 | 6 | Yes | Omphalitis, fever, recurrent respiratory tract infections | No | No | *-* | 21984 | Expired |
| LAD1^o^ | P13 | ♂ | 2 | 4.5 | Yes | Omphalitis, perianal rash | No | NA | *Klebsiella sp, S. aureus* | 10899 | Expired |
| LAD1^o^ | P14 | ♂ | 0.03 | 26.4 | Yes | Omphalitis, skin rash, otitis media, diarrhea, pneumonia, gluteal abscess, perianal fistula, hepatosplenomegaly | No | No | *E. coli, Pseudomonas aeruginosa* | 24475 | Expired |
| LAD1- | P15 | ♂ | 4 | 78 | No | otitis media, bronchopneumonia, lesions on chin, cough, fever, Non healing ulcers, splenomegaly | No | No | *-* | 16136 | Lost to follow up |
| LAD1^o^ | P16 | ♂ | 0.1 | 1.6 | Yes | loose stool, septicemia, aeruginosa region abscess, recurrent ulcers, perianal fistula | No | No | *-* | 142900 | Expired |
| LAD1^o^ | P17 | ♀ | 0.4 | 6 | Yes | laryngotracheobronchitis, pneumonia, pallor, pyoderma, mild hepatomegaly, post inflammatory hypo pigmentation | Yes | Yes | *Pseudomonas aeruginosa* | 78256 | Expired |
| LAD1^o^ | P18 | ♂ | 0.5 | 1 | Yes | umbilical sepsis with discharge | Yes | NA | *Klebsiella Sp* | 72900 | Expired |
| LAD1^o^ | P19 | ♀ | 1 | 7 | Yes | perianal rash, Budd Chiari syndrome | No | No | *-* | 48848 | Expired |
| LAD1**^+^** | P20 | ♂ | 24 | 83 | No | Recurrent Skin lesion all over the body with itching, fever | Yes | Yes | *Gram negative bacilli* | 19320 | Alive |
| LAD1**^+^** | P21 | ♀ | 36 | 83 | No | Recurrent cutaneous infections affecting various parts of the body, multiple deep scars of healed cutaneous infections | No | Yes | *-* | 7500 | Lost to follow up |
| LAD1^o^ | P22 | ♀ | 0.23 | 7 | No | Diarrhea, lymphadenopathy, skin blisters, dermatitis with cellulitis, umbilical sepsis twice | Yes | Yes | *Citrobacter and Staphylococcus aureus* | 31000 | BMT Alive |
| LAD1^o^ | P23 | ♂ | 1.64 | 2 | NA | perianal ulcers, splenomegaly, tachycardia | No | NA | *Non fermenting gram negative bacteria, P. aeruginosa* | 52830 | Lost to follow up |
| LAD1^o^ | P24 | ♀ | 0.5 | 1.5 | Yes | perianal, eye infection, Non healing ulcers at multiple places | No | No | *-* | 124310 | Expired |
| LAD1**^+^** | P25 | ♂ | 0.7 | 3.5 | No | Recurrent ulcerative lesion involving perianal region, lower limbs | No | No | *Pseudomonas, coliform* | 73467 | Lost to follow up |
| LAD1**^+^** | P26 | ♂ | 48 | 132 | No | Recurrent skin ulcers, cyanosis, | No | Yes | *Pseudomonas aeruginosa* | 43000 | Alive |
| LAD1**^+^** | P27 | ♀ | 132 | 168 | No | Pyoderma gangrenosum | No | No | *-* | 11000 | Alive |
| LAD1**^+^** | P28 | ♂ | 48 | 144 | No | Pyoderma gangrenosum | Yes | No | *AFB, fungus* | 19670 | Alive |
| LAD1**^+^** | P29 | ♂ | 36 | 192 | NA | multiple vesicular lesions, ulcer, pyoderma gangrenosum on thigh | Yes | No | *-* | 24800 | Lost to follow up |
| LAD1^o^ | P30 | ♂ | 5 | 8 | NA | Fever, fungal rash in groin, buccal and mucosa | No | No | *Pseudomonas aeruginosa, Fungal* | 32550 | Expired |

| **Phenotype** | **Patient** | **Gender** | **Age of onset (months)** | **Age of Diagnosis (months)** | **Umbilical Cord Separation** | **Other clinical presentations** | **Family History** | **Consanguinity** | ***Organisms Isolated*** | **ANC** | **Outcome** |
| --- | --- | --- | --- | --- | --- | --- | --- | --- | --- | --- | --- |
| LAD1^o^ | P31 | ♂ | 0.03 | 6 | No | Omphalitis, B/l ear discharge, fever, abdominal distention | Yes | Yes | *-* | 78561 | Alive |
| LAD1^o^ | P32 | ♀ | 0.5 | 1 | No | Recurrent respiratory tract infection | Yes | No | *-* | 21102 | Alive |
| LAD1^o^ | P33 | ♀ | 0.9 | 3 | Yes | Omphalitis, Multiple skin lesions, Otitis, ecthyma gangrenosum | Yes | Yes | *Pseudomonas aeruginosa* | 37352 | Expired |
| LAD1^o^ | P34 | ♂ | 0.2 | 3 | Yes | Omphalitis, ulcer over left axilla and neck, fever redness over body, cold ear discharge | No | Yes | *Pseudomonas aeruginosa* | 103272 | Expired |
| LAD1^o^ | P35 | ♂ | 1.6 | 2.23 | Yes | Non-pyogenic aeruginosa inflammation, ulceration | No | No | *Pseudomonas aeruginosa, Staphylococcus aureus* | 42700 | Alive |
| LAD1^o^ | P36 | ♂ | 0.03 | 3 | No | Fever, skin rashes, Omphalitis | No | No | *-* | 28249 | Expired |
| LAD1**^+^** | P37 | ♂ | 0.5 | 3 | Yes | perianal infection, fever, sepsis, Omphalitis | No | No | *Acinetobacter* | 59810 | Expired |
| LAD1^o^ | P38 | ♂ | 0.03 | 1.3 | Yes | Omphalitis, pallor, pyoderma | Yes | Yes | *Stingomonas paucimobilis* | 39827 | Expired |
| LAD1^o^ | P39 | ♂ | 1 | 6 | Yes | Omphalitis, otitis, fever, Respiratory tract, urinary tract, | No | Yes | *Klebsiella sp., enterococcus, Candida, Pseudomonas sp.* | 27398 | Expired |
| LAD1^o^ | P40 | ♂ | 0.03 | 4 | No | Pneumonia, Omphalitis | No | No | *-* | 23679 | Lost to follow up |
| LAD1**^+^** | P41 | ♂ | 2 | 5 | Yes | Pneumonia, Omphalitis | No | Yes | *-* | 90869 | Expired |
| LAD1o | P42 | ♂ | 0.03 | 3 | No | Gingivitis, Non healing ulcers | No | No | *-* | 24570 | Expired |
| LAD1- | P43 | ♂ | 2.5 | 4.5 | Yes | Omphalitis, Recurrent infections | No | No | *-* | 74064 | Expired |
| LAD1^o^ | P44 | ♀ | 0.03 | 1 | Yes | perianal excoriation, periumbilical erythema | No | No | *-* | 51348 | Expired |
| LAD1^o^ | P45 | ♂ | 3 | 5 | No | cold fever vomiting, Omphalitis, gingivitis | No | No | *-* | 86016 | Expired |
| LAD1- | P46 | ♂ | 0.13 | 2 | Yes | Fever, sepsis, poor feeding | Yes | No | *Acinetobacter baumannii, Pseudomonas aeruginosa* | 23155 | Expired |
| LAD1^o^ | P47 | ♀ | 1 | 48 | No | Fever, recurrent infection | No | No | *-* | 14856 | Expired |
| LAD1^o^ | P48 | ♀ | 0.03 | 0.56 | No | Fever, Omphalitis | No | No | *Pseudomonas aeruginosa* | 49910 | Expired |
| LAD1^o^ | P49 | ♂ | 0.6 | 0.7 | No | Omphalitis, mild hepatosplenomegaly, submucosal edema involving terminal ileum, perianal rash recurrent diarrhea | No | No | *-* | 63999 | Expired |
| LAD1^o^ | P50 | ♂ | 1 | 1.5 | Yes | Omphalitis, otitis media | No | Yes | *Staphylococcus aureus* | 18240 | Expired |
| LAD1^o^ | P51 | ♂ | 0.1 | 2 | Yes | Otitis media, skin and soft tissue infection | Yes | Yes | *-* | 20604 | Expired |
| LAD1^o^ | P52 | ♂ | 0.1 | 41 | Yes | Omphalitis, fever, recurrent respiratory tract infections | Yes | Yes | *-* | 11766 | Lost to follow up |
| LAD1^o^ | P53 | ♀ | 0.03 | 2 | Yes | Omphalitis, fever, sepsis, poor feeding | Yes | Yes | *-* | 22230 | Expired |
| LAD1^o^ | P54 | ♀ | 3 | 4 | Yes | Omphalitis, Non healing ulcer on lower limbs, sepsis, hepatosplenomegaly | Yes | Yes | *-* | 19417 | Expired |
| LAD1^o^ | P55 | ♂ | 1.15 | 2.5 | Yes | Otitis media with mastoiditis | No | Yes | *Pseudomonas aeruginosa* | 71721 | Alive |
| LAD1^o^ | P56 | ♂ | 1.84 | 10 | Yes | Omphalitis, fever, loose stool, gingivitis | No | Yes | *-* | 43520 | Lost to follow up |
| LAD1^o^ | P57 | ♂ | 1.15 | 2.5 | Yes | Ear discharge, scalp seborrhea, ulcer on lower limbs | Yes | Yes | *Pseudomonas aeruginosa* | 71721 | Expired |
| LAD1^o^ | P58 | ♂ | 0.2 | 1.5 | Yes | Omphalitis, Necrotic Ulcer on lower limbs | No | Yes | *-* | 116160 | Expired |
| LAD1^o^ | P59 | ♀ | 0.32 | 2 | No | Fever, recurrent respiratory tract infections | Yes | Yes | *-* | 19904 | Expired |
| LAD1^o^ | P60 | ♂ | 0.7 | 1 | Yes | Fever, skin rash | No | Yes |  | 25574 | Lost to follow up |
| LAD1^o^ | P61 | ♀ | 2 | 3 | No | Fever, recurrent respiratory tract infections | No | Yes | *-* | 94506 | Lost to follow up |
| LAD1^o^ | P62 | ♂ | 2 | 42 | Yes | Omphalitis, pyoderma | No | Yes | *Klebsiella sp., Proteus, Enterococcus* | 79866 | Expired |
| LAD1**^+^** | P63 | ♂ | 2 | 3 | No | Fever, cough and cold , skin rash, marginal hepatomegaly | No | Yes | *-* | 60451 | Expired |
| LAD1^o^ | P64 | ♂ | 0.16 | 0.42 | No | Pneumonia, skin rashes | No | No | *Klebsiella pneumonia* | 79866 | Expired |
| LAD1^o^ | P65 | ♀ | 1.5 | 6 | Yes | Pneumonia, gingivitis | No | Yes | *-* | 19325 | Expired |
| LAD1**-** | P66 | ♂ | 0.1 | 1 | Yes | Omphalitis, fluctuant swelling around umbilicus with discoloration of overlying skin | No | Yes | *-* | 29250 | Expired |
| **Phenotype** | **Patient** | **Gender** | **Age of onset (months)** | **Age of Diagnosis (months)** | **Umbilical Cord Separation** | **Other clinical presentations** | **Family History** | **Consanguinity** | ***Organisms Isolated*** | **ANC** | **Outcome** |
| LAD1**-** | P67 | ♀ | 0.12 | 3 | Yes | Omphalitis, Fever, recurrent infection multiple swelling all over abdomen | No | Yes | *Pseudomonas aeruginosa* | 25000 | Alive |
| LAD1**-** | P68 | ♀ | 2 | 10 | Yes | Recurrent loose stool and oral thrush, corneal ulcer in left eye, omphalitis | No | Yes | *-* | 23586 | Expired post BMT |
| LAD1^o^ | P69 | ♂ | 0.1 | 40 | Yes | Recurrent fever, Omphalitis |  | Yes | *-* | 42000 | Expired |
| LAD1^o^ | P70 | ♀ | 12 | 192 | No | Recurrent fever, loose motions, Omphalitis |  | Yes | *-* | 56000 | Expired |
| LAD1^o^ | P71 | ♀ | 0.16 | 0.42 | No | Pustule at multiple sites | No | No | *Klebsiella pneumoniae* | 37128 | Expired Post BMT |
| LAD1^o^ | P72 | ♂ | 0.23 | 6.5 | Yes | Ecthyma gangrenosum, perianal ulcers, gum swelling | No | Yes | *-* | 32350 | Expired |
| LAD1^o^ | P73 | ♀ | 0.23 | 4 | No | Omphalitis, otitis media, culture proven sepsis | No | Yes | *Staphylococcus aureus, Pseudomonas aeruginosa* | 44940 | Expired |
| LAD1^o^ | P74 | ♀ | 0.33 | 7 | Yes | Omphalitis, ulcers, otitis media, | No | Yes | *Pseudomonas aeruginosa* | 45135 | Expired |
| LAD1^o^ | P75 | ♀ | 0.23 | 2 | No | fever, loose stool cough | NA | NA | *-* | 35336 | Expired |
| LAD1^o^ | P76 | ♂ | 0.23 | 2 | Yes | Omphalitis, sepsis, pneumonia, perianal erythematous lesion | Yes | No | *-* | 53488 | Expired |
| LAD1^o^ | P77 | ♀ | 0.03 | 0.23 | Yes | C/o fever, cold & cough, recurrent LRTI, Bronchopneumonia, multiple admissions for same, omphalitis. Pallor, Hepatosplenomegaly , B/L Pleural effusion, abdominal wall edema, Abscesses, Intestinal perforation with peritonitis Skin - Impetigo contagiosa | No | Yes | Pus - *MRSA,* Blood C/S *- Klebsiella Pneumoniae* | 91600 | Expired |
| LAD1^o^ | P78 | ♀ | 0.13 | 1 | No | Neonatal sepsis, omphalitis USG - Bulky renal Parenchyma, Fever, LRTI, Stool - occult blood | No | Yes | *Gram positive cocci, ET C/S:* Stenotrophomonas, *Enterobacter sp.* | 60810 | Alive |
| LAD1^o^ | P79 | ♂ | 0.13 | 1 | No | pyogenic meningitis, omphalitis, recurrent loose stools | No | No | *Bacterial* | 6500 | Alive |
| LAD1- | P80 | ♀ | 7 | 10 | No | H/o infection, LRTI shadow on XRC | No | Yes | *-* | 34140 | Alive |
| LAD1- | P81 | ♂ | 0.23 | 2 | - | fever, cold recurrent infections |  | Yes | *-* | 37800 | Lost to follow up |
| LAD1^o^ | P82 | ♀ | 6 D | 2 | Yes | Pyoderma gangrenosum and Pseudomonas aeruginosa, Multiple skin ulcers (Non healing) | No | Yes | *-* | 117700 | Alive |
| LAD1^o^ | P83 | ♀ | 9 D | 0.2 | Yes | Meningitis and recurrent infections | No | No | *Klebsiella Pneumoniae* | 87800 | Expired |
| LAD1^o^ | P84 | ♂ | 0.5 | 4 | Yes | Recurrent infection omphalitis | Yes | Yes | *-* | 56700 | Alive |
| LAD1^o^ | P85 | ♀ | 0.3 | 0.5 | Yes | Recurrent infection, omphalitis | No | Yes | *Pseudomonas aeruginosa* | 43400 | Expired |
| LAD1^o^ | P86 | ♂ | 0.23 | 1 | No | Recurrent infection, omphalitis, skin and subcutaneous Non healing ulcers | No | Yes | *Staphylococcus* | 28000 | Expired Post HSCT |
| LAD1^o^ | P87 | ♀ | 0.23 | 4 | No | Recurrent infections, omphalitis, kin and subcutaneous Non healing ulcers | No | Yes | *Staphylococcus* | 25000 | Alive post HSCT |
| LAD1^o^ | P88 | ♀ | 0.23 | 1 | No | Recurrent infection, omphalitis, skin and subcutaneous Non healing ulcers | Yes | Yes | *Staphylococcus* | 40000 | Alive post HSCT |
| LAD1^o^ | P89 | ♂ | 0.23 | 1 | No | Recurrent infection. Omphalitis, skin and subcutaneous Non healing ulcers | No | Yes | *Staphylococcus* | 34000 | Alive post HSCT |
| LAD1^o^ | P90 | ♂ | 0.4 | 5 | No | Umbilical sepsis, omphalitis, recurrent admission for fever, irritability, URTI | Yes | Yes | *No* | 39280 | Alive post HSCT |
| LAD1^o^ | P91 | ♂ | 0.23 | 1.3 | Yes | Fever, omphalitis, Necrotizing otitis externa, urinary tract Infections | No | No | *Pseudomonas,* gram negative Bacilli, *E-coil.* | 108300 | Expired |
| LAD1^o^ | P92 | ♀ | 3 | 8 | Yes | B/L otitis media, Pneumonia | No | Yes | *-* | 81900 | BMT Alive |
| LAD1^o^ | P93 | ♂ | 0.7 | 4 | Yes | Skin abscesses, right ear discharge, omphalitis | Yes | Yes | *-* | 65000 | Expired |
| LAD1^o^ | P94 | ♀ | 3 | 12 | - | Recurrent ear infections - 10 episodes, ulcers over aeruginosa area | Yes | No | *-* | 128540 | BMT Alive |
| LAD1^o^ | P95 | ♂ | 1 | 7 | Yes | Omphalitis for 3 months, recurrent diarrhea, sepsis, perianal ulcer | Yes | Yes | *-* | 120000 | BMT Alive |
| LAD1^o^ | P96 | ♀ | 1 | 3 | Yes | Ear discharge | Yes | Yes | *-* | 130000 | Lost to follow up |
| LAD1^o^ | P97 | ♂ | 0.7 | 12 | - | Recurrent infections Multiple cutaneous lesions, Periodontitis | Yes | Yes | *Staphylococcus aureus* | 35200 | Alive |
| LAD1^o^ | P98 | ♂ | 1 | 24 | - | Recurrent infections Multiple cutaneous lesions, Periodontitis | Yes | Yes | *Staphylococcus aureus* | 42600 | Alive |
| LAD1^o^ | P99 | ♂ | 0.07 | 36 | - | Recurrent infections Multiple cutaneous lesions, Periodontitis | No | NS | *Staphylococcus aureus, Enterococcus faecalis* | 61000 | Alive |
| LAD1^o^ | P100 | ♂ | 0.03 | 1 | Yes | Fever, skin rash, oral infection | No | No | *Pseudomonas, E. coli* | 41560 | Lost to follow up |
| **Phenotype** | **Patient** | **Gender** | **Age of onset (months)** | **Age of Diagnosis (months)** | **Umbilical Cord Separation** | **Other clinical presentations** | **Family History** | **Consanguinity** | ***Organisms Isolated*** | **ANC** | **Outcome** |
| LAD1^o^ | P101 | ♂ | 36 | 60 | NA | recurrent fever, large skin ulcers with black eschar like scab on it | Yes | Yes | *Aspergillus niger* | 65000 | Expired |
| LAD1^o^ | P102 | ♀ | 0.03 | 1 | NA | Fever, recurrent infection skin rash | No | No | NA | 87000 | Lost to follow up |
| LAD1^o^ | P103 | ♂ | 132 | 12 | NA | Recurrent infection | Yes | Yes | *Staphylococcus aureus* | 35200 | Alive |
| LAD1- | P104 | ♂ | 72 | 36 | NA | Cystic swelling which ruptures, scaly papery thin skin | No | No | *Staphylococcus aureus* | 61000 | Alive |
| LAD1^o^ | P105 | ♂ | 0.5 | 3.3 | Yes | Recurrent infections | No | No | *K. pneumonia, C. albicans, P. aeruginosa* | 125000 | Expired |
| LAD1^o^ | P106 | ♂ | 0.52 | 0.33 | No | Skin rash, recurrent infection | No | No | *Klebsiella pneumonia-* Carbapenem resistant | 70500 | Expired |
| LAD1^o^ | P107 | ♀ | 0.26 | 1.5 | NA | Pyogenic meningitis with brain abscess, facial palsy, pharyngeal weakness with panniculitis, subcutaneous fat atrophy, swelling in perianal region, inability to close eye, multiple ulcerative lesion, yellowish white discharge from umbilicus, lethargic, abdominal distention and dilated veins, exposure keratopathy, polymorphonuclear leukocytosis | Yes | No | *E. coli, Pseudomonas aeruginosa* | 126000 | Alive |
| LAD1- | P108 | ♀ | 0.32 | 5 | Yes | LAD Type I with Budd Chiari Syndrome with very severe pneumonia, sepsis, periumbilical redness with yellowish purulent discharge, lower limb edema, progressing to abdomen and face, pallor, abdominal distension, lethargy, excessive crying and decreased activity, hepatosplenomegaly with coarsed liver, anasarca, neutrophilia, gross ascites, chronic liver parenchymal disease with portal distension | No | No | *E. coli* | 30000 | Expired |
| LAD1^o^ | P109 | ♂ | 0.2 | 1 | Yes | fever, loose stools, cough, oral ulcers, oral thrush, umbilical cord not fallen, A deep perianal ulcer, failure to thrive, posterior arching of head and a perianal ulcer at admission, umbilical redness without discharge | Yes | No | *Staphylococcus, Pseudomonas* | 145200 | Lost to follow up |
| LAD1^o^ | P110 | ♀ | 0.1 | 6 | Yes | Omphalitis, nodule like lesion in perianal area, recurrent febrile episodes associated with coryza, difficulty in feeding/excessive crying, neutrophilic leukocytosis, inflammatory markers, borderline hepatomegaly | Yes | No | *NA* | 55620 | Lost to follow up |
| LAD1^o^ | P111 | ♂ | 0.32 | 5 | Yes | Recurrent pyogenic infections, ear discharge (yellow green), chronic suppurative otitis media, facial nerve palsy, redness and purulent discharge from umbilicus, delayed fall of umbilical cord, swelling and redness following intramuscular injection, development of abscess over right thigh, umbilical hernia, ulcers, CXR - some right sided infiltrates shown, Aspiration, tachypnea, respiratory distress | Yes | Yes | *Pseudomonas aeruginosa CMV* | 50000 | Lost to follow up |
| LAD1^o^ | P112 | ♂ | 0.5 | 5.5 | Yes | Omphalitis, fever, irritability, frequent loose stools and non healing perianal excoriation, periumbilical region redness, abdominal distension, recurrent diarrhea, pneumonia, thrombocytopenia, umbilical sepsis, anemia, borderline hepatosplenomegaly, rapid breathing, edema, very severe pneumonia | No | No | *Enterococcus faecium (Sensitive to gentamycin, vancomycin, etc.), P. aeruginosa (sensitive to ciprofoxin, etc.)* | 69200 | Lost to follow up |
| LAD1^o^ | P113 | ♂ | 0.09 | 2 | NA | Umblical sepsis, abscess at intramuscular injection site, acute gastroenteritis, ASOM in left ear, infection around genitalia and recurrent oral ulcers (Candida), groin ulcers, swelling of both limbs & progressed up to hips swelling in scrotum, swelling on face, pneumonia, chronic inflammation in colon, renal parenchymal disease, fungal diarrhea, oral thrush, sepsis, juvenile periodontitis, repeated skin infection, URI, hepatosplenomegaly, persistent neutrophilic leukocytosis, pallor, clubbing, skin sepsis, recurrent GIT, lymphadenopathy, anal and mucosal ulcers, pyoderma, white patch on face, patchy alopecia, blackening of teeth, acute suppurative otitis media | No | No | *Staphylococcus aureus sepsis, Candida, Ring worm, yeast* cells occasional | 69700 | Expired |
| LAD1^o^ | P114 | ♂ | 0.82 | 96 | No | Pyoderma gangrenosum, recurrent respiratory tract infections, multiple cutaneous ulcers, scaly lesions over face, multiple hypo pigmented papules present over face, neck, chest, etc., ostopenia - steroid toxicity, admitted for ulceration with micro abscess and granulomatous inflammation, multiple skin lesion on lower limbs, mild hepatosplenomegaly, necrotizing inflammation, vesicular lesions, edema, deep fungal infection | Yes | No | Methiliclin resistant *Staphylococcus aureus - Pyoderma gangrenosum, Coliform* | 30000 | Expired |
| **Phenotype** | **Patient** | **Gender** | **Age of onset (months)** | **Age of Diagnosis (months)** | **Umbilical Cord Separation** | **Other clinical presentations** | **Family History** | **Consanguinity** | ***Organisms Isolated*** | **ANC** | **Outcome** |
| LAD1^o^ | P115 | ♂ | 5 | 5 | - | Omphalitis, urachal cyst, swelling & redness & pus discharge from umbilicus, umbilical -enteric fistula with non-healing ulcers over abdomen, dilated veins and fecal discharge | Yes | - | *NA* | 80190 | Alive post BMT |
| LAD1^o^ | P116 | ♀ | 4 | 5 | - | Recurrent infection, skin rash, pneumonia | - | - | *-* | 61420 | Lost to follow up |
| LAD1^o^ | P117 | ♂ | 0.23 | 2 | - | Pneumonia, skin and soft tissue infections | - | - | *-* | 45201 | Expired |
| LAD1^o^ | P118 | ♀ | 0.23 | 1 | - | Recurrent infection |  | - | *-* | 84763 | Lost to follow up |
| LAD1^o^ | P119 | ♂ | 0.23 | 0.3 | - | Neutrophilic leukocytosis, Fever and excessive cry | - | No | *Blood culture CONS positive,* | 81000 | Lost to follow up |
| LAD1- | P120 | ♂ | 7 | 14 | Yes | Ulcer over left thigh, boil over left gluteal region, pus discharge from ulcer, fever, single fissure present over groin, multiple discrete skin colored umbilical papules, hypo pigmented scar, edema | - | No | *Klebsiella Pneumonia* | 38600 | Lost to follow up |
| LAD1^o^ | P121 | ♀ | 2.5 | 3 | No | Periumbilical erythema, persistent redness around umbilicus, minimal discharge from umbilicus | - | Yes | *E. coli* | 38150 | Lost to follow up |
| LAD1^o^ | P122 | ♂ | 0.23 | 0.3 | No | Recurrent infection omphalitis | - | NS | Not isolated | 167000 | Lost to follow up |
| LAD1^o^ | P123 | ♀ | 0.16 | 11 | NA | Ulcer over palate (whitish discharge), regurgitation of feeds from nose, palatable perforation, neutrophilic leukocytosis, delayed shedding of umbilical stump | - | No | *Staphylococcus, Pseudomonas* | 61020 | Lost to follow up |
| LAD1^o^ | P124 | ♂ | 24 | 72 | Yes | Recurrent episodes of Fever, fluid filled vesicles, periumbilical erythema with mucoid discharge, 2-3 black eschar (5-6mm) around neck and trunk, recurrent non suppurative infections, omphalitis | - | Yes | *MRSA* | 45000 | Lost to follow up |
| LAD1^o^ | P125 | ♂ | 0.5 | 2 | No | Swelling and redness around umbilicus, necrotic patch in anterior side of neck which initially started as 2 large vesicles, Erythema around umbilicus, omphalitis, necrotizing fasciitis, neutrophilic leukocytosis | - | No | *Pseudomonas aeruginosa* | 150000 | Lost to follow up |
| LAD1^o^ | P126 | ♀ | 0.62 | 5 | NA | Fluid filled vesicles over left thigh with mild surrounding erythema, fever, poor feed and lethargy, peeling of skin on trunk with erythema, history of loose stools, necrotizing fasciitis, No healing of wound, necrotic wound over left thigh, splenomegaly, moderate ascites, pleural effusion, sepsis, staphylococcal skin scalded syndrome, fluid filled vesicles over whole body, abdominal distension, neutrophilic leukocytosis, ulcer, fever, pallor, desquamated rash, papulo pustular lesions in face, erosive blister like lesion on right foot, umbilical hernia, | - | Yes | *Actinobacter baumannii, Staphylococcus hominis, Enterobacter sp., Rodemia, yeast +++* | 57000 | Expired |
| LAD1^o^ | P127 | ♂ | 1 | 3 | Yes | Recurrent, Upper respiratory tract infection, ear discharge, right finger pulp infection, Ulceration upper chest without pus | - | None | NA | 87900 | Alive |
| LAD3 | P128 | ♀ | 1 | 2 | No | Neonatal sepsis, Bluish rash, Pin point [petechial](https://www.google.com/search?safe=active&sxsrf=ALeKk01Z6RJ_VPXqymWfwKrVrF2CMUP24g:1607065257783&q=petechial&spell=1&sa=X&ved=2ahUKEwiNpcjO4LPtAhWDaCsKHdtZCXEQkeECKAB6BAgREDA) rash over face, limbs trunk. | Yes | Yes | NA | 99610 | Alive |
| LAD3 | P129 | ♂ | 0.03 | 12 | Yes | Abscesses, cytopenia, bleeding from mouth, Skin rash all over the body | No | No | NA | 35490 | Expired |
| LAD3 | P130 | ♂ | 0.66 | 120 | No | Glanzmann like thrombasthenia, gastrointestinal tract infection | Yes | No | NA | 26200 | Alive |
| LAD3 | P131 | ♂ | 1 | 168 | No | Glanzmann like thrombasthenia, urinary tract infection, gastrointestinal tract infection, colitis | Yes | - | *E. coli* | 31700 | Alive |
| LAD3 | P132 | ♂ | 9 | 54 | No | recurrent ear infection, gum and skin bleeds | - | No | NA | 30000 | Alive |
|  |  |  |  |  |  |  |  |  |  |  |  |
| #Note : ♂ = male; ♀ = female | | |  |  |  |  |  |  |  |  |  |
| NA- No data available | |  |  |  |  |  |  |  |  |  |  |
