# Supplementary material for: Clinical and Genetic Spectrum of a Large Cohort of Patients With Leukocyte Adhesion Deficiency Type 1 and 3: A Multicentric Study From India
Source: Front Immunol. 2020 Dec 16;11:612703. doi: 10.3389/fimmu.2020.612703 (PMC7772426; doi:10.3389/fimmu.2020.612703)
Supplement: Supplementary Table S2 — Mutation spectrum of ITGβ2 gene and FERMT3 genes. [file Table_2.docx]

| **Gene** | **Affected Domain** | **Nucleotide Change** | **Amino Acid Substitution** | **Exon/ Intron** | **Type of Mutation** | **Patient** | **Genbank Accession no./ References** |
| --- | --- | --- | --- | --- | --- | --- | --- |
| ***ITGβ2*** | PSI integrins | c.212_212delG | p.Gly72Alafs*32 | Exon 4 | Deletion | P54 | MT488444 |
|  | VWFA | c.977_979delGTG | p.Val327del | Exon 8 | Deletion | P55 | MT488445 |
|  | Mid | c.1225-4_1268del48 | p.Ile409Alafs*23 | Exon 11 | Deletion | P76 | Novel |
|  | CRR | c.1472_1475delAGGA | p.Gln491Argfs*37 | Exon 12 | Deletion | P69 | MT488447 |
|  | CRR | c.1798_1798delG | p.Val600Tyrfs*33 | Exon 13 | Deletion | P56 | MT488446 |
|  | CRR | c.1516delT | p.Cys506Alafs*23 | Exon 12 | Deletion | P123 | Novel |
|  | VWFA | c.1057_1062delins38 | p.Val353Serfs*4 | Exon 9 | Frame shift delins | P1,P2 | CX152213 |
|  | VWFA | c.557_558insA | p.Leu187Alafs*78 | Exon 6 | Insertion | P57 | MT488448 |
|  | VWFA | c.942_943insAACA | p.Ile316Lysfs*11 | Exon 8 | Insertion | P58 | MT488449 |
|  | VWFA | c.812_822insA | p.Leu275Alafs*39 | Exon 7 | Insertion | P111 | Novel |
|  | VWFA | c.945_946insAAAC | p.Ile316Lysfs*39 |  | Insertion | P118 | Novel |
|  | PSI integrin | c.322C>T | p.Arg108Ter | Exon 4 | Nonsense | P25, P47, P77 | BM1528049, rs772862268 |
|  | VWFA | c.393T>A | p.Tyr131Ter | Exon 5 | Nonsense | P48 | MT488450 |
|  | VWFA | c.562C>T | p.Arg188Ter | Exon 6 | Nonsense | P22, P30,P71#,P75#, P120, P125 | rs148877937, CM065288 |
|  | VWFA | c.658G>T | p.Glu220Ter | Exon 6 | Nonsense | P5,P6,P24,P49,P50 | CM151554 |
|  | Mid region | c.1264C>T | p.Gln422Ter | Exon 11 | Nonsense | P19 | Novel |
|  | CRR | c.1590C>G | p.Tyr530Ter | Exon 12 | Nonsense | P51 | CM120006 |
|  | CRR | c.1632C>G | p.Tyr544Ter | Exon 12 | Nonsense | P3,P4# | CM120007 |
|  | Integrin β tail | c.1866T>A | p.Cys622Ter | Exon 13 | Nonsense | P52 | MT488451 |
|  | Integrin β tail | c.1888G>T | p.Glu630Ter | Exon 14 | Nonsense | P18, P53 | BM1537113 |
|  | CRR | c.2055C>A | p.Try685Ter | Exon 14 | Nonsense | P107# | Novel |
|  | CRR | c.2077C>T | p.Arg693Ter | Exon 14 | Nonsense | P121,P122, P115, P116.P117 | Novel |
|  | CRR | c.1840G>T, | p.Glu614Ter | Exon 13 | Nonsense | P79, P109 | Novel |
|  | PSI integrin | c.106T>A | p.Cys36Ser | Exon 3 | Missense | P31 | CM120009 |
|  | VWFA | c.382G>T | pAsp128Tyr | Exon 5 | Missense | P8, P15, P94# | rs137852615, CM109171 |
|  | VWFA | c.505G>A | p.Gly169Arg | Exon 6 | Missense | P9,P33,P34 | rs137852612,CM900146 |
|  | VWFA | c.533C>T | p.Pro178Leu | Exon 6 | Missense | P10,P35,P36,P66,P67,P71#,P75#, P90, P92 | rs137852614, CM930445 |
|  | VWFA | c.616C>T | p.His206Tyr | Exon 6 | Missense | P42 | MT366558 |
|  | VWFA | c.710T>G | p.Leu237Arg | Exon 6 | Missense | P43 | MT36655 |
|  | VWFA | c.715G>A | p.Ala239Thr | Exon 6 | Missense | P32 | rs179363873, CM109170 |
|  | VWFA | c.725A>G | p.Gln242Arg | Exon 6 | Missense | P44 | MT488438 |
|  | VWFA | c.751G>A | p.Gly251Ser | Exon 7 | Missense | *P23#P63#,P72#, P73#, P74#* | rs179363873 |
|  | VWFA | c.756G>C | p.Trp252Cys | Exon 7 | Missense | P45,P107# | MT488439 |
|  | VWFA | c.758G>A | p.Arg253His | Exon 7 | Missense | P47 | MT488443 |

| **Gene** | **Affected Domain** | **Nucleotide Change** | **Amino Acid Substitution** | **Exon/ Intron** | **Type of Mutation** | **Patient** | **Genbank Accession no./ References** | |  |
| --- | --- | --- | --- | --- | --- | --- | --- | --- | --- |
| ***ITGβ2*** | VWFA | c.809C>T | p.Ala270Val | Exon 7 | Missense | P21 | rs147318988 | |  |
|  | VWFA | c.817G>A | p.Gly273Arg | Exon 7 | Missense | P12,P13#, P38,P39,P40,P41, P94# | rs137852618, CM990754 | |  |
|  | VWFA | c.850G>A | p.Gly284Ser | Exon 7 | Missense | P7, P11,P19,P37 | rs137852616, | |  |
|  | VWFA | c.953C>A | p.Pro318His | Exon 8 | Missense | P68 | MT488440 | |  |
|  | VWFA | c.962C>A | p.Ala321Glu | Exon 8 | Missense | P17 | CM151555 | |  |
|  | Mid Region | c.1283T>G | p.Ile428Ser | Exon 11 | Missense | P72# | MT488441 | |  |
|  | CRR | c.1621T>C | p.Cys541Arg | Exon 12 | Missense | P46 | MT366556 | |  |
|  | CRR | c.1745G>A | p.Cys582Tyr | Exon 13 | Missense | P76# | Novel | |  |
|  | CRR | c.1777C>T | p.Arg593Cys | Exon 13 | Missense | P16 | rs137852609, CM900148 | |  |
|  | CRR | c.1828C>A | p.Pro610Thr | Exon 13 | Missense | P4# | MT488442 | |  |
|  | TM | c.2147G>T | p.Gly716Val | Exon 15 | Missense | P74# | Novel | |  |
|  | CRR | c.1768 T>C | p.Cys590Arg | Exon 13 | Missense | P114 | Novel | |  |
|  | - | c.329-2A>G | IVS4-2 | Intron 4 | Splice site | P70 | Novel | |  |
|  | VWFA | c.499+1G>T | IVS5+1 | Intron 5 | Splice site | P59 | Novel | |  |
|  | VWFA | c.500+1G>A | IVS5+1 | Intron 5 | Splice site | P119 | Novel | |  |
|  | VWFA | c.742-1G>A | IVS6-1 | Intron 6 | Splice site | P60,P61, P73# | Novel | |  |
|  | VWFA | c.897+1G>A | IVS7+1 | Intron 7 | Splice site | P14,P62 | CS920765 | |  |
|  | VWFA | c.897+1G>C | IVS7+1 | Intron 7 | Splice site | P13# | CS151558 | |  |
|  | VWFA | c.1084+4A>G | IVS9+4 | Intron 9 | Splice site | P124 | Novel | |  |
|  | - | c.1224+4A>G | IVS10+4 | Intron 10 | Splice site | P20,P26,P27,P28,P29 | CS151559 | |  |
|  | - | c.1658-2A>G | IVS12-2 | Intron 12 | Splice site | P63 | CS120036 | |  |
|  | - | c.1878-2A>C | IVS13-2 | Intron 13 | Splice site | P23,P64 | CS151557 | |  |
|  | - | c.1878-1G>A | IVS13-1 | Intron 13 | Splice site | P65 | Novel | |  |
|  |  |  |  |  |  |  |  | |  |
| ***FERMT3*** | - | c.305T>C | p.Leu102Pro | Exon 3 | Missense | P128 | Novel | |  |
|  | PH | c.1343G>A | p.Trp448Ter | Exon 12 | Nonsense | P129 | Novel | |  |
|  | FERM | c.687G>T | p.Trp229Cys | Exon 6 | Missense | P130, P131 | Novel | |  |
|  | FERM | c.821A>G | p.Gln274Arg | Exon 7 | Missense | P132 | Novel | |  |
|  |  |  |  |  |  |  | |  | |
|  | Note: # heterozygous, PSI- plexins, semaphorins, and integrins domain, VWFA- Von Willebrand factor type A domain, TM- transmembrane, CRR- cysteine rich repeat region, PH- Pleckstrin homology domain | | | | | | | | |
